# Plasma membrane calcium ATPases and cerebellar pathology: what’s the role in the ataxia?

**DOI:** 10.1186/s13062-025-00702-2

**Published:** 2025-11-06

**Authors:** Caterina Peggion, Ivan Marchionni, Ernesto Carafoli, Marisa Brini, Tito Calì

**Affiliations:** 1 Department of Biology, University of Padova, Padova, Italy; 2 Department of Biomedical Sciences (DSB), University of Padova, Padova, Italy; 3 Veneto Institute of Molecular Medicine, Padova, Italy; 4 Department of Pharmaceutical and Pharmacological Sciences, University of Padova, Padova, Italy; 5 Study Center for Neurodegeneration (CESNE), University of Padova, Padova, Italy; 6 Padova Neuroscience Center (PNC), University of Padova, Padova, Italy

**Keywords:** Ca^2+^ signaling, Plasma membrane calcium ATPases, Cerebellar ataxia, Ca^2+^ microdomains.

## Abstract

Ca²⁺ signaling is essential for neuronal development, migration, synaptic activity, spine plasticity, neurotransmitter release, membrane excitability, and long-term synaptic plasticity, as well as for the coupling between membrane depolarization and downstream signaling. Traditionally, Plasma Membrane Ca²⁺ ATPases (PMCAs) were considered high-affinity, low-capacity calcium extruders. However, recent evidence reveals that the PMCA-Neuroplastin complex facilitates ultrafast Ca²⁺ clearance at kilohertz frequencies, reshaping our understanding of calcium regulation, in particular in neurons. For bulk Ca²⁺ clearance, they are overshadowed by more powerful low-affinity/high-capacity systems on the plasma membrane. This raises key questions: what is the specific physiological and pathological role of PMCAs? Why do cells require a high-affinity/low-capacity, ATP-dependent extrusion mechanism? What is the functional meaning of the diversity of isoforms (four) and splice variants (over thirty)? And why do neurons localize distinct PMCA pumps to pre- and postsynaptic sites? The prevailing hypothesis is that PMCAs fine-tune Ca²⁺ microdomains through local regulation and interactions with specific protein partners. Finally, understanding their role in Purkinje cells (PCs) is particularly relevant, as alterations in PMCA function have been implicated in cerebellar pathology and ataxia.

## Clinical trial number

Not applicable.

## Introduction

### Neuronal Ca^2+^ homeostasis: mechanisms of regulation and the pivotal role of plasma membrane Ca²⁺-ATPases (PMCAs)

**Fig. 1:**
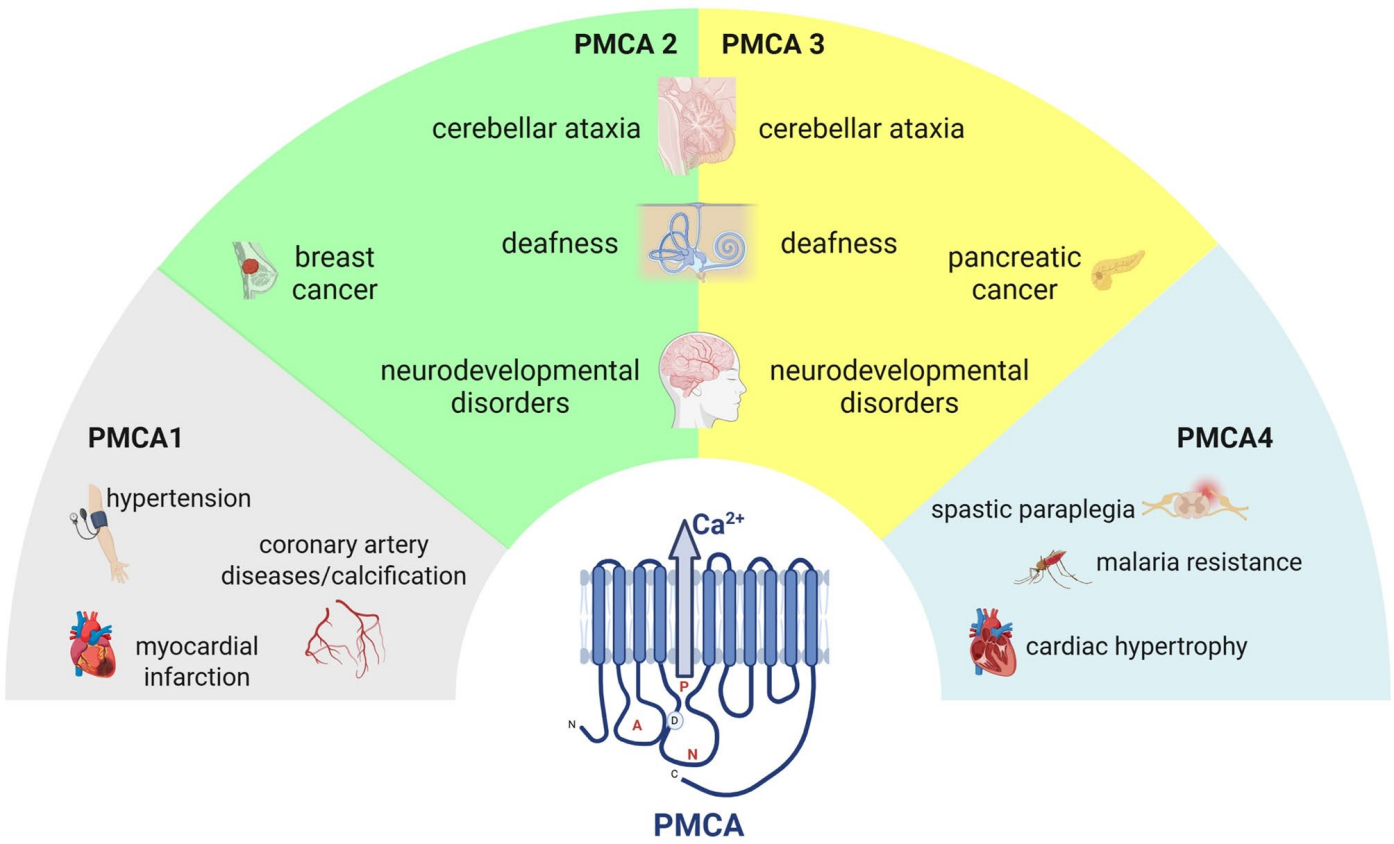
Disorders related to dysfunctional PMCA1–4 isoforms. The 2D structure of the PMCA pump is shown at the bottom center. The four isoforms and pathologies related their mutations—PMCA1 (grey), PMCA2 (green), PMCA3 (yellow), and PMCA4 (light-blue)—are arranged in a semi-circular layout and connected to the central structure

Intracellular calcium (Ca²⁺) signaling is one of the most versatile regulatory systems in neurons, acting as a critical second messenger in processes such as neurotransmitter release, gene transcription, synaptic plasticity, metabolism, and cell survival or death [1, 2]. Due to its central role in neuronal physiology and the potential cytotoxicity associated with Ca²⁺ overload, neurons have evolved highly efficient and spatially compartmentalized mechanisms for maintaining Ca²⁺ homeostasis [3–7]. At rest, the intracellular concentration of free Ca²⁺ in neurons is tightly maintained at ~ 100 nM, whereas the extracellular concentration is around 1–2 mM, generating a large electrochemical gradient. This gradient enables rapid Ca²⁺ entry in response to membrane depolarization or receptor activation. Key pathways for Ca²⁺ influx include voltage-gated Ca²⁺ channels (VGCCs), which open in response to membrane depolarization; ionotropic glutamate receptors such as NMDA and AMPA [8] receptors, which are activated by presynaptic glutamate release; and members of the transient receptor potential (TRP) channel family, which mediate Ca²⁺ entry in response to various stimuli including temperature, osmolarity, and ligand binding. Following Ca²⁺ entry, neurons employ multiple buffering and clearance strategies to restore basal Ca²⁺ levels and prevent the activation of deleterious pathways. These include Ca²⁺-binding proteins (e.g., calbindin, calretinin, parvalbumin [9]) that transiently sequester free Ca²⁺, as well as organelle-based uptake systems like the endoplasmic reticulum Ca²⁺-ATPases (SERCAs) and mitochondrial Ca²⁺ uniporter (MCU), which temporarily store Ca²⁺ within internal compartments. A critical component of the Ca²⁺ clearance machinery is the extrusion of Ca²⁺ across the plasma membrane, primarily mediated by two systems: the Na⁺/Ca²⁺ exchanger (NCX) and the plasma membrane Ca²⁺-ATPase (PMCA) [10]. NCX operates with low affinity but high capacity, making it suitable for rapid Ca²⁺ clearance following large influxes. For a long time, it was thought that PMCAs function with high affinity and low capacity, allowing for the precise fine-tuning of Ca²⁺ levels, particularly during periods of low-level or sustained Ca²⁺ signaling. However, recent studies demonstrated that PMCA2 pump act as an ultrafast Ca^2+^ extrusion system, and that is particularly important in the brain where signal transmission occurs at high frequency of up to 1 kHz [11]. PMCAs are P-type ATPases [12] that utilize the energy from ATP hydrolysis to actively transport Ca²⁺ ions out of the cytoplasm and into the extracellular space, typically at a 1:1 stoichiometry. Four PMCA isoforms (PMCA1–4) are expressed in mammals [13, 14], with multiple splice variants [15], conferring cell-type-specific regulatory properties and localization, additionally, a long C-terminal cytosolic tail which is the site of interaction with most of the regulatory factors of the pump (the most important being calmodulin) is a key feature [16–19]. Recently, it was demonstrated that native PMCAs are heteromers constituted of PMCA1-4 proteins and the Ig-domain containing protein Neuroplastin (NPTN) and/or Basigin that represent obligatory auxiliary subunit of PMCA complexes useful for their stability, trafficking, reliable expression in the plasma membrane and proper Ca^2+^ transport [20, 21]. Although PMCA2 and PMCA3 are traditionally considered neuron-specific [22], all four isoforms (PMCA1–4) are expressed in neurons. These PMCAs are differentially distributed in subcellular compartments, including synaptic terminals and dendritic spines, where they help shape local Ca²⁺ transients. PMCA1 and PMCA4 are enriched in the cortex and hippocampus but are less prominent in the cerebellum. In contrast, PMCA2 and PMCA3 are highly expressed in the cerebellum, PMCA2 predominantly at postsynaptic sites and PMCA3 at presynaptic terminals, particularly in granule cells (mainly a and b splice variants) [22], cerebellar glomeruli, and Purkinje fiber terminals [23]. Both isoforms are also present in PCs somata and dendrites, as well as in GABAergic neurons of the molecular and granule layers, with expression levels correlating with neuronal maturation and synaptogenesis [22, 24]. These distribution patterns are mirrored in the pathophysiology of PMCA-related disorders: dysfunction in PMCA2 and PMCA3 cannot be compensated by the more widely expressed but less active PMCA1 and PMCA4 (Fig. 1). Additionally, PMCA2 is also particularly notable for its high basal activity and rapid response to Ca²⁺ transients, even in the absence of calmodulin (CaM), making it especially effective in neurons exposed to frequent and large Ca²⁺ fluctuations [25–27]. It also displays weak CaM-dependent activation, but much faster activation kinetics compared to PMCA1 and PMCA4. Similarly, PMCA3 shows strong activity at low basal Ca²⁺ levels and shares the high CaM affinity of PMCA2, in the range of 5–10 nM (as characterized in the truncated ‘a’ variant) [28]. PMCA activity is also subject to regulation by calmodulin and phosphorylation, allowing dynamic modulation in response to cellular signaling states. Importantly, PMCAs contribute not only to global Ca²⁺ homeostasis but also to the regulation of Ca²⁺ microdomains, which are essential for the specificity of downstream signaling cascades [29]. Another crucial regulator of PMCA activity/pumping velocity is its partner NPTN, that by complexing with the pump under physiological condition, contributes to determine the high efficiency and speed (within ms-windows) of Ca^2+^ extrusion and its ability to operate at cycle rates in the KHz-range (similar to those reported for NCX2). Such data are profoundly different from previous results demonstrating values of about 50–100/s that could be due to the usage of different experimental settings or approaches [11]. Their precise control over Ca²⁺ decay kinetics ensures the fidelity of synaptic responses, prevents Ca²⁺ spillover between signaling pathways, and protects neurons from excitotoxic damage associated with prolonged Ca²⁺ elevation. In summary, while multiple mechanisms cooperate to regulate neuronal Ca²⁺ dynamics, PMCAs occupy a uniquely strategic position in maintaining long-term Ca²⁺ homeostasis and in refining the spatial and temporal resolution of Ca²⁺ signals. Their role is especially vital in neurons, where even subtle disturbances in Ca²⁺ handling can lead to profound functional deficits and contribute to the pathogenesis of neurodegenerative diseases [30]. In addition to isoform-specific differences, variations in binding partners and Ca²⁺-regulated/dependent local factors may further contribute to the distinct disease phenotypes associated with PMCA dysfunction (see below).

### Ca^2+^ signaling in the cerebellum: physiological roles and pathological implications

The cerebellum is a highly organized structure composed of different classes of excitatory and inhibitory neurons and orchestrates a wide range of essential tasks, related to sensorimotor integration, precision in voluntary movement, postural control, motor learning [31, 32]. It also contributes to certain higher-order processes such as attention, language, and emotion regulation [33, 34]. These diverse functions rely on the integrity of its highly stereotyped microcircuitry and the temporal precision of its neuronal patterns [33], both of which are critically dependent on the proper regulation of intracellular Ca²⁺. Within the cerebellum, Purkinje cells (PCs) represent the principal integrative neurons and the sole output of the cerebellar cortex. These cells exhibit elaborate dendritic arbors and receive two major excitatory inputs: parallel fibers (PFs) (from granule cells) and climbing fibers (CFs) (from the inferior olive). Both forms of input trigger distinct patterns of Ca²⁺ influx in PCs dendrites [35]. Climbing fiber stimulation, in particular, produces large and widespread Ca²⁺ transients, which are essential for the induction of long-term depression (LTD [36]) —a form of synaptic plasticity thought to underlie motor learning. Calcium entry in these neurons occurs via multiple routes, including voltage-gated Ca²⁺ channels (especially P/Q-type), NMDA receptors, and intracellular release through IP₃- and ryanodine-sensitive pathways. In such a highly active environment, Ca²⁺ clearance mechanisms must operate with both speed and precision to ensure that signaling remains spatially and temporally confined. PMCAs, especially isoforms PMCA2 and PMCA3, are abundantly expressed in PCs and play a fundamental role in restoring Ca²⁺ to basal levels after synaptic activation in physiological conditions [23]. Their high affinity for Ca²⁺ makes them particularly suited to maintaining Ca²⁺ homeostasis during sustained or repetitive firing, when intracellular Ca²⁺ remains elevated for prolonged periods. Furthermore, PMCA distribution within PCs is not uniform; these pumps are often localized in specific membrane subdomains, suggesting specialized roles in controlling Ca²⁺ microdomains essential for local signaling cascades. Disruptions in cerebellar Ca²⁺ handling might have severe functional consequences. Impaired PMCA activity, whether due to genetic mutations, age-related decline, oxidative stress, or pathological hyperexcitability, can result in aberrant intracellular Ca²⁺ accumulation, triggering maladaptive plasticity, mitochondrial dysfunction, and activation of pro-apoptotic pathways. In murine models, mutations in PMCA2 (such as the *wriggle mouse sagami*) result in ataxia, Purkinje cell degeneration, and impaired motor learning, highlighting the non-redundant role of PMCAs in cerebellar function [37, 38]. Similarly, deficiencies in Ca²⁺-buffering proteins or excessive Ca²⁺ influx through mutated VGCCs have been linked to spinocerebellar ataxias and other neurodegenerative conditions with cerebellar involvement [39, 40]. Indeed, Ca²⁺ dysregulation in the cerebellum does not only affect motor systems. There is increasing evidence that disturbances in cerebellar Ca²⁺ homeostasis may contribute to cognitive and neuropsychiatric symptoms, possibly through altered cerebellar-thalamo-cortical communication [41]. This expands the potential clinical relevance of PMCA function beyond classical motor disorders. Therefore, Ca²⁺ signaling in the cerebellum is not only a driver of fast synaptic events but also a gatekeeper of long-term neuronal viability and network adaptability. PMCAs serve as critical modulators in this context, ensuring that Ca²⁺ signals are timely and tightly regulated to support both normal cerebellar operations and long-term cellular health. Their dysfunction, by contrast, can tip the balance toward pathological states, making them promising targets for therapeutic intervention in cerebellar and broader neurological disorders. Interestingly, dysfunction of PMCA is not only linked to neurological disorders, but its partner protein NPTN, previously recognized for its role in plasticity-dependent synaptic restructuring [42], also plays a crucial part. Dysfunction or deficiency of NPTN, which is known to affect PMCA surface expression and activity, has been associated with impaired synaptic transmission and deficits in associative learning and memory. Given that NPTN interacts with and modulates PMCA function, this connection provides clearer insight into how these two proteins together influence synaptic processes and cognitive functions in the brain [43].

### Anatomical and functional roles of Purkinje cells

**Fig. 2:**
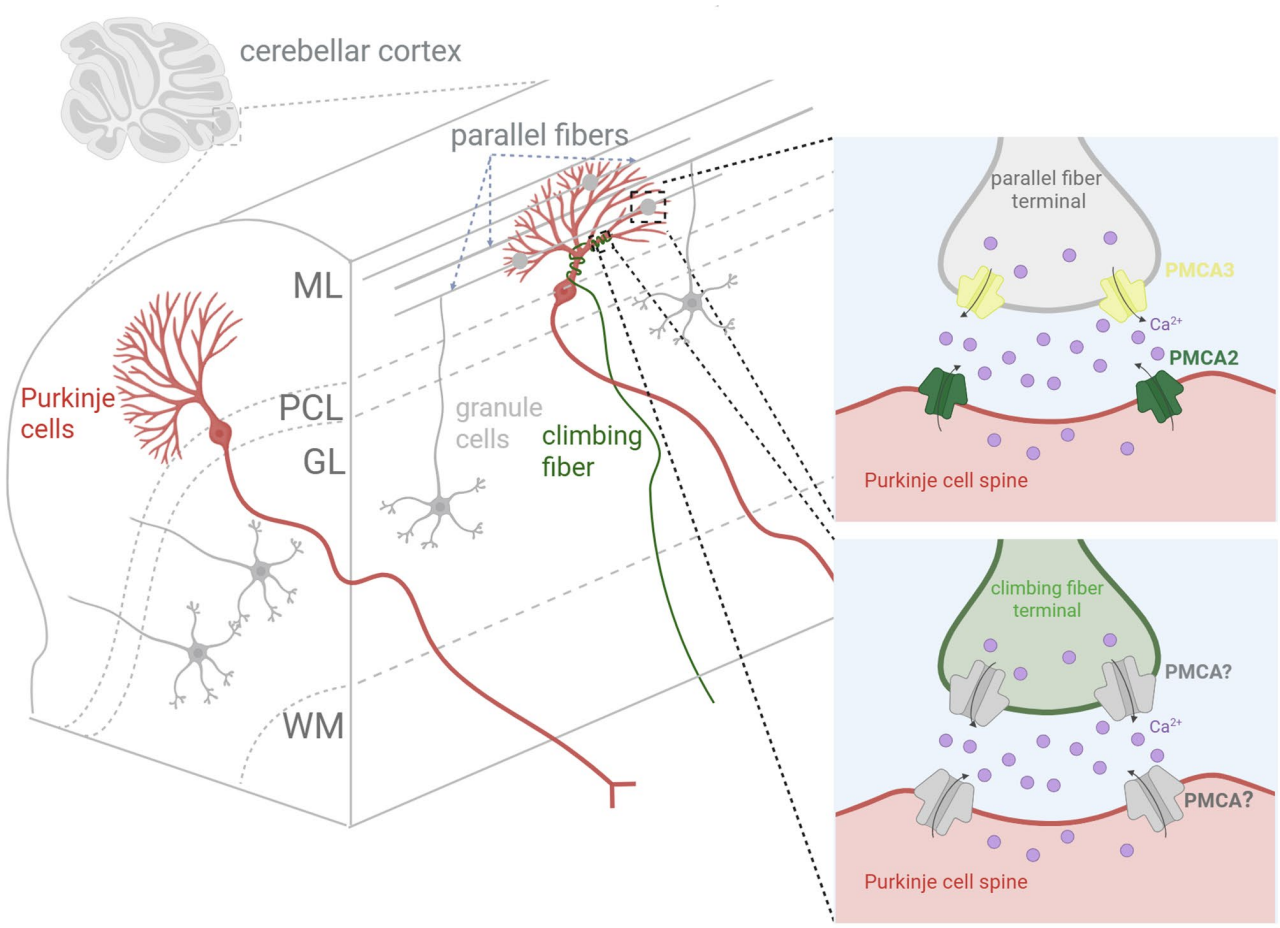
Schematic overview of cerebellar circuitry. PFs (grey) and CFs (green) are shown. Cartoon representation of a cerebellar section with increasing magnification across its layers. On the right-top, a zoom-in of the pre-synaptic PF onto Purkinje cell (PC) dendrite; on the right-bottom, the pre-synaptic climbing fiber onto PC dendrite are shown. The distribution of PMCA isoforms at pre- and post-synaptic sites is also illustrated. Note the isoform composition at the CFs-PCs is still not known (?). Abbreviations: ML, molecular layer; PCL, Purkinje Cell Layer; GL, Granule Cell Layer; WM, white matter

PCs represent the cornerstone of cerebellar cortical architecture and function. Anatomically, they are among the largest and most morphologically complex neurons in the central nervous system. Their soma is located in the Purkinje cell layer, positioned between the molecular layer above and the granule cell layer below [35, 44–47]. The hallmark of Purkinje cell anatomy is a highly elaborate, fan-shaped dendritic arbor that extends into the molecular layer in a two-dimensional plane. This arrangement enables extensive synaptic integration within a tightly organized cerebellar framework. Each Purkinje cell receives excitatory input from two distinct long-range axons: PFs and CFs. PFs are the axons of cerebellar granule cells, and they form synapses along the distal dendrites of PCs. A single Purkinje cell can receive input from over 100,000 PFs, providing a dense, diffuse signal that encodes diverse sensory and motor information [48]. In contrast, each Purkinje cell is innervated by a single climbing fiber, originating from the contralateral inferior olivary nucleus [35]. Despite the singular input, CFs form numerous powerful synapses on the proximal dendritic tree, capable of generating large, complex spikes and robust intracellular Ca²⁺ transients. Functionally, PCs serve as the sole output neurons of the cerebellar cortex. They project inhibitory (GABAergic) axons to the deep cerebellar nuclei (DCN), which in turn send excitatory output to various premotor and motor areas of the brain [35] (Fig. 2). PCs exhibit two primary types of action potential firing: simple spikes, generated by synaptic input from PFs and modulated by intrinsic pacemaking activity (20–100 Hz) [49–52]; and complex spikes, elicited by climbing fiber activation, and accordingly activating motor areas via disinhibition of the DCN [53–56]. By modulating the activity of the DCN, PCs regulate the timing and coordination of voluntary movements, fine-tune motor commands, participate in the learning of motor skills [53, 57, 58] and possibly, at the level of higher cognitive functions such as memory, attention and visuospatial abilities (for review see [33]). The influx of Ca²⁺ through voltage-gated Ca²⁺ channels and the release from internal stores following climbing fiber activation initiates cascades critical for synaptic plasticity. Calcium-dependent activation of kinases (e.g., protein kinase C) and phosphatases underlie the molecular basis of LTD, which is believed to be a cellular correlate of motor learning. Given the high frequency of synaptic input and the complex intracellular Ca²⁺ dynamics involved, PCs are heavily reliant on precise Ca²⁺ buffering and extrusion mechanisms. As anticipated above, PMCAs (1–4) are widely expressed in neurons, and PMCA2 and 3 are the isoform most highly expressed in the cerebellum [59]. Interestingly, PMCA 2 and 3 are mainly co-expressed at the distal dendrites of PCs, on the other hand the other two isoform (PMCA1 and 4) are largely present at the presynaptic level in the granule cells layer [60]. This suggests that all four isoforms contribute at different levels and in specific manners at the level of cerebellar microcircuit PFs, controlling specifically the GCs-PCs synaptic circuit. On the other hand, low level of PMCAs (1–4) are expressed in other cerebellar circuits [59]. This specific and localized expression of the different isoforms of PMCAs suggest a key role in controlling the homeostatic level of Ca^2+^ only at PF synapses (see Fig. 2). The dysregulation of Ca^2+^ homeostasis at these synapses might cause an altered output signal carried by the PCs highlighting the crucial role of PMCAs in maintaining proper signaling and behaviour. In pathological conditions, PCs are particularly vulnerable to Ca²⁺ dysregulation. Due to their high metabolic demand and Ca²⁺ sensitivity, they are often the first neuronal population to exhibit degeneration in cerebellar ataxias [61], ischemia, or excitotoxic injury. Very recently, using various techniques such as immunogold electron microscopy, SDS-digested freeze-fracture replicas, and quantitative mass spectrometry in brain neurons, it was demonstrated that the density of PMCAs on the dendrites of PCs is approximately 250/µm², and about 50/µm² in the active zones of parallel fiber presynaptic terminals. This study showed that PMCAs are the most abundant Ca²⁺ transporters in the brain, surpassing the amounts of both NCX and SERCA. The discovery that PMCA2-NPTN-mediated Ca²⁺ extrusion occurs on the millisecond timescale and represents the most abundant Ca²⁺ clearance mechanism has crucial implications for the temporal dynamics of Ca²⁺ signaling. This is especially important in the central nervous system, where rapid removal of excess intracellular Ca²⁺ and prevention of its accumulation are essential for reliable, timely, and accurate neurotransmission at dendrites and synapses [11].

### Purkinje cell: the role of intracellular Ca^2+^ in physiological and pathological conditions

The proper functioning of PCs is crucial for normal cerebellar activity, and any disruption or dysregulation in their firing rates can have significant consequences for executive functions, emotional processing, and social cognition [62, 63]. Altered action potential firing rates of the PCs in the cerebellum have been associated with various disorders, including ataxia and autism spectrum disorders (ASD) [63, 64]. Studies of ischemic or traumatic damages to specific cerebellar micro-zones have shown specific deficits in cognitive, emotional and perception processing [65].

Nevertheless, detailed insights into the intrinsic and extrinsic circuit dysfunction are still needed to understand the role of specific neuronal microcircuitry and long-range specific inputs that underlie cerebellum functions and dysfunctions. Calcium plays a central role in this process. VGCCs, particularly P/Q-type channels, are crucial for initiating Ca²⁺ influx during depolarization at the presynaptic level. The spatial and temporal characteristics of Ca²⁺ transients are finely controlled through Ca²⁺-binding proteins (such as calbindin), intracellular sequestration (via endoplasmic reticulum and mitochondria), and active extrusion mechanisms, most notably the plasma membrane Ca²⁺-ATPases (PMCAs). Calcium accumulation resulting from excessive influx or impaired extrusion may lead to abnormal excitability. In some cases, this manifests as hyperexcitability, with irregular or burst-like firing in PCs. In other cases, prolonged Ca²⁺ elevation could activate Ca²⁺-dependent potassium channels (such as BK channels [66]), leading to membrane hyperpolarization that silences spontaneous activity for extended periods, affecting the functional activity of PCs. In both cases, local and long-range brain structures might be affected by the altered activity of the PCs. For instance, the disruption of Ca²⁺ transients might alter synaptic plasticity destabilizing the proper role of these synapses leading to deficits in motor learning. Furthermore, sustained intracellular Ca²⁺ elevation may activate apoptotic signaling pathways, contributing to cellular degeneration, a hallmark of many cerebellar ataxias. Interestingly, pathological conditions can also involve insufficient intracellular Ca²⁺ signaling. This may occur due to excessive Ca²⁺ clearance, overexpression of buffering proteins, or impaired Ca²⁺ influx. In such cases, failure to reach critical Ca²⁺ thresholds prevents the activation of key intracellular pathways necessary for plasticity and normal firing modulation. This can result in poorly timed or unsynchronized output, even in the absence of overt Ca²⁺ overload. In both extremes, whether Ca²⁺ levels are too high or too low, the result is a disruption in the temporal precision and reliability of PCs firing. In conclusion, intracellular homeostasis of Ca²⁺ plays a pivotal role in shaping the excitability and firing patterns of PCs (Fig. 2). Mntaining Ca²⁺ balance is essential and crucial not only for supporting physiological firing and synaptic plasticity but also for preventing degeneration. Disruption of this balance, through altered Ca²⁺ entry, buffering, or extrusion, can profoundly compromise PC function and underlie various forms of cerebellar dysfunction. Structural abnormalities or degeneration of PCs typically result in impaired motor coordination, ataxia, tremor, and deficits in motor learning, highlighting their indispensable role in cerebellar function [67, 68]. In summary, PCs are anatomically intricate and functionally indispensable neurons that serve as the principal computational and inhibitory output units of the cerebellar cortex. Their integrative properties, synaptic plasticity mechanisms, and dependence on Ca²⁺ dynamics make them essential for the cerebellum’s role in motor coordination and adaptive learning.

### PMCAs and diseases

**Fig. 3:**
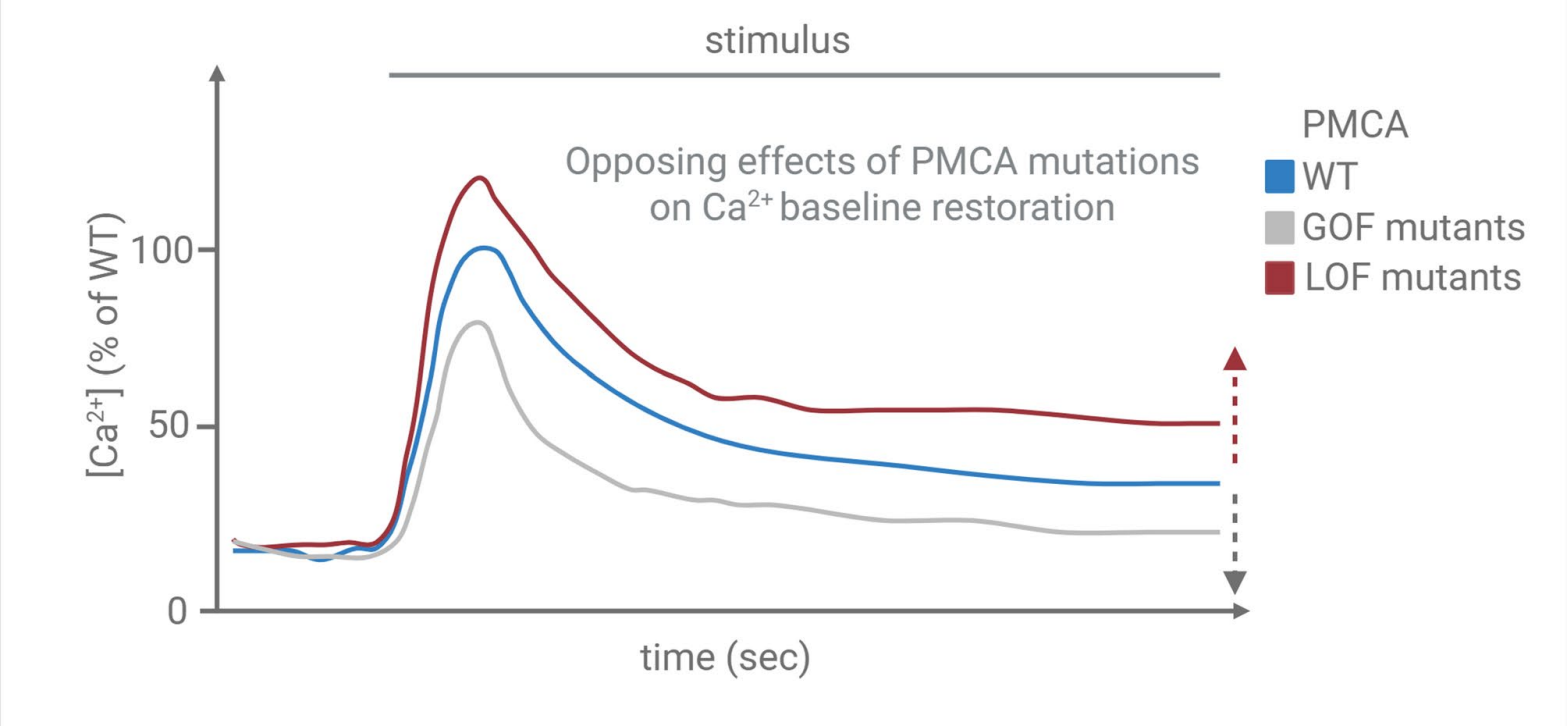
Effect of PMCA on the calcium extrusion. The calcium transient following stimulation in WT (blue), gain of function (GOF; red), and loss of function (LOF; grey) mutations of PMCAs is shown. Specifically, GOF and LOF mutations result in altered kinetics of cytosolic calcium concentration over time

As already mentioned, it is widely recognized that mutations in genes coding the different PMCA isoforms are related to diverse human diseases (Fig. 1). Such correlation was strongly established by genome-wide association studies (GWAS). For example, common SNPs located in the human *ATP2B1* gene (12q21 chromosome region), which encodes the ubiquitous PMCA1 pump, were found to be associated with blood pressure variation (both systolic and diastolic), development of hypertension and resistance to blood pressure medical treatment [69–74]. Also, PMCA1 mutations were found to be linked to an increased risk for coronary artery disease [75], myocardial infarction [76], salt sensitivity and coronary artery calcification in chronic kidney disease [76, 77]. Several SNPs within the *ATP2B4* gene (human chromosome 1q32) linked PMCA4 with resistance to severe forms of falciparum malaria in numerous patients from Africa, Asia and Oceania [78–83]. PMCA4 was also associated with familial spastic paraplegia (FSP) neurological disorders since its heterozygous missense mutation (c.803G >A, p.R268Q) has been found in a Chinese family affected by the disease [84]. The mutation causes a reduced maximal Ca²⁺ increase after KCl-induced depolarization suggesting a close link between functional changes in Ca²⁺ homeostasis and the pathology [85]. In mice, the complete ablation of PMCA4 was shown to lead to sperm motility defects and male infertility [13, 14] as a consequence of either increased NOS activity, NO, and OONO − levels or mitochondrial Ca^2+^ accumulation, impaired mitochondrial activity and reduced ATP levels. Other studies performed in PMCA4 KO mice also demonstrated that the pump governs myocardial growth, cardiac hypertrophy and peripheral vascular tone [86–92]. PMCA2 SNPs were linked instead to autism spectrum disorders (ASDs). Such linkage is strongly corroborated by the existence of an altered Ca^2+^ signaling in autism, generally involving mutations that either increase Ca^2+^ influx or amplify intracellular Ca^2+^ signaling [93]. Interestingly, independent GWAS identified a link between the chromosome region 3p25 (in which also PMCA2 is located) and autism [94–96]. This highlights the key role played by neuronal PMCA2 in the development/function of the nervous system by regulating Purkinje cell morphology, cerebellar cortex development or synapse biogenesis. As already mentioned, PMCA2 has a limited tissue distribution. It is also expressed in the mammary glands during lactation (in both rodents and humans) [97], where it is involved in the excretion of Ca²⁺ ions into milk. In addition to its physiological function in the mammary gland, PMCA2 expression was reported to be increased in breast cancer by interacting with the epidermal growth factor receptor 2 (HER2) and promoting its higher expression at the cell surface [98]. Regarding PMCA3, a role in pancreatic cancers has been revealed, being the T543M missense mutation, located between the catalytic Asp and the ATP binding site important for the disease [99]. Somatic mutations of ATP2B3 gene were also found to be associated with aldosterone-producing adenoma (APA) [100], characterized by higher levels of aldosterone secretion [100–103]. Interestingly, most of the mutations found are in the M4 transmembrane domain which is involved in Ca^2+^ binding and ion (Fig. 3).

Mutations and function impairment of PMCA pumps were also unequivocally linked to deafness in mice [27, 104–106] and humans [107, 108], strongly demonstrating the essential role of PMCA2 in the inner ear and linking the deafness phenotype to defects in its Ca²⁺ extruding functionality.

### Mastering calcium: PMCA’s central role

In resting neurons, at low intracellular Ca^2+^ the C-terminal tail of the PMCA interacts with the main body of the protein keeping it in an autoinhibited state. It does so by interacting with two sites in the first main cytoplasmic domain of the pump [109] and one in the second, close to the catalytic site [110, 111]. Local Ca^2+^ increase activates calmodulin that removes the C-terminal tail from the inhibitory sites. Two moments are of key importance: (i) under basal, unstimulated conditions, they safeguard the low cytosolic Ca^2+^ levels (≈100 nM) from unwanted changes by promptly sensing even subtle increases in [Ca^2+^]_i_; (ii) following stimulation, as cytosolic [Ca²⁺] rapidly declines from the micromolar range (≈ 5–10 µM) toward basal nanomolar levels, primarily driven by the robust activity of the NCX, the PMCA gradually takes over, becoming functionally engaged as [Ca²⁺] falls to approximately 1 µM to 500 nM acting locally within defined subcellular microdomains to help fine-tune Ca²⁺ clearance. PMCA binds Ca²⁺ ions with high affinity (Km of 100–200 nM) but, in particular isoforms 1 and 4 operate at a relatively slow turnover rate (10–150 s⁻¹). In contrast, the Na⁺/Ca²⁺ exchanger (NCX) exhibits low affinity but high capacity, with a Kcat of approximately 2500 s⁻¹ and a 1 Ca²⁺/3 Na⁺ exchange ratio. As a result, PMCA is particularly effective at detecting and removing Ca²⁺ even when intracellular concentrations are very low, making it well-suited for maintaining basal cytosolic Ca²⁺ levels in the nanomolar range. Calmodulin binding significantly enhances PMCA function by increasing the Ca²⁺ binding affinity of the pump 20- to 30-fold. This highly cooperative activation mechanism renders PMCAs extremely sensitive to small fluctuations in intracellular Ca²⁺ concentration ([Ca²⁺]_i_), allowing precise regulation of Ca²⁺ homeostasis under resting conditions. An important yet often overlooked aspect of CaM-mediated regulation is its inherently oscillatory nature. As intracellular Ca²⁺ levels rise to a threshold that promotes CaM binding, the pump is relieved from autoinhibition and begins actively extruding Ca²⁺. This, in turn, reduces local Ca²⁺ concentrations, leading to the dissociation of CaM from its binding site. Once CaM detaches, the autoinhibitory domain re-engages, halting pump activity [112]. Therefore, CaM-induced activation of the pump is not sustained, but instead occurs in rapid, transient bursts. The PMCA pumps are therefore low abundant membrane proteins with a high affinity to Ca^2+^ enabling them to finely regulate cellular Ca²⁺ homeostasis when the intracellular [Ca^2+^] exactly falls within a very specific and narrow range with an upper and lower limit imposed by the PMCA isoform/splice variant present in each cell type and cell sub-compartment (Fig. 3). This is achieved through additional regulation/activation not only by calmodulin, but also by acidic phospholipids (essentially, phosphatidylserine) [113–117], polyunsaturated fatty acids, oligomerization [118], calpain [119], caspases [120] or by phosphorylation [121–123]. The pump also binds Ca^2+^ with varying affinities at sites upstream and downstream of the CaM binding domain [124], the effects of these other allosteric Ca^2+^ binding sites are not yet known.

Therefore, it is likely to hypothesize that their key role is in the temporal regulation of Ca^2+^ in specific sub-plasma membrane compartments/microdomains [125–128] which influences locally several Ca^2+^-dependent enzymes and/or Ca^2+^- or voltage gated channels of the plasma membrane as well as Ca^2+^-independent interacting partners that are relevant to neuronal function [88, 129, 130]. A number of genetic mutations of PMCA pumps are associated with pathological phenotypes, those of the neuron-enriched are linked to cerebellar ataxias (the role of PMCA2 in deafness will not be discussed here, see [131] for excellent reviews on the topic). Biochemical analysis of the mutated pumps overexpressed in model cells have revealed their impaired Ca²⁺ export function. The defect in the bulk cytosolic Ca^2+^ homeostasis is minor, in keeping with the above-mentioned role of local control of plasma membrane Ca^2+^ microdomains.

### Mutations of the neuronal enriched PMCA2 and PMCA3 are linked to cerebellar ataxia

PMCA2 is particularly abundant in the cell bodies, dendrites and dendritic spines of cerebellar PCs [59, 132] where it rapidly clears the Ca^2+^ inputs received from PFs [59, 132, 133], thus explaining also why both PMCA2-null and deafwaddler 2 J (dfw2J) mice [27, 106, 134] manifest cerebellar pathology, resulting not only in deafness, as previously discussed, but also in motor deficits and ataxia. The relationship between PMCA2 and cerebellar dysfunction was also confirmed by a gene expression profiling study on lymphoblasts from autistic and nonaffected sib pairs demonstrating a link between altered PC morphology, cerebellar development, or synapse biogenesis and the differential expression of such pump [135].

The first pure missense mutation in PMCA2 pump related to an exclusively ataxic phenotype was the V1143F variant (located in the CaM-BD), over a cadherin 23 wt background, found in a subject affected by congenital cerebellar ataxia with the hearing ability fully retained, thus confirming the fact that defects of the PMCA2 pump isoform might be a direct cause of an ataxic phenotype [136]. Molecular dynamics studies demonstrated that such mutation impairs the binding of CaM-BD to calmodulin and consequently is responsible for an impaired Ca^2+^ extrusion capacity of the pump [136].

**Fig. 4:**
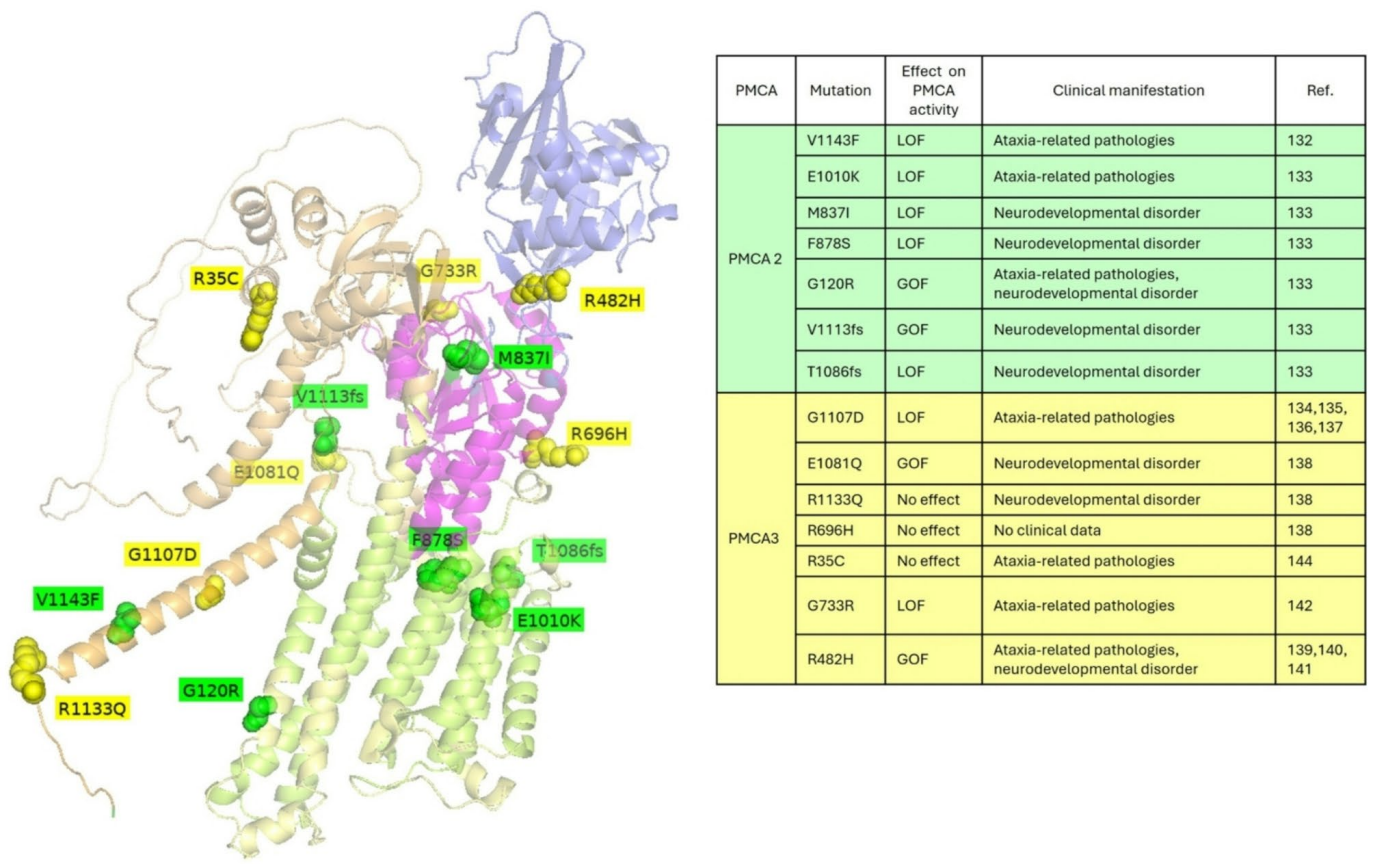
Position of disease-related mutations in the 3D structure of human PMCA. The different domains are colored as follows: transmembrane domains in lemon yellow, A-domain in wheat yellow, P-domain in purple, and N-domain in light blue. Mutated amino acids in PMCA2 or PMCA3 are shown in green and yellow, respectively. On the right, a table summarizes the missense mutations. Their effect on PMCA activity (GOF and LOF), their associated pathologies are reported in the third and fourth column, respectively. The structure was generated using PyMOL, starting from the AlphaFold model AF-Q01814-F1-v4

Very recently, a group of deleterious heterozygous ATP2B2 missense and end-truncating variants were found in 7 patients affected by a variable spectrum of neurological or neurodevelopmental disorders, including dystonia, ataxia, intellectual disability, behavioral symptoms, and seizures [137] (Fig. 2). Such variants, of which six are confirmed as de novo mutation, comprised 5 missense substitutions in evolutionarily conserved sites (i.e., 3028G >A, p.(Glu1010Lys); c.2511G >T, p.(Met837Ile); c.2633T >C, p.(Phe878Ser); c.358G >A, p.(Gly120Arg) and c.358G >C, p.(Gly120Arg)) and 2 frameshift variants (i.e., c.3338_3339del, p.(Val1113Glyfs∗36) and c.3254dup, p.(Thr1086Aspfs∗64)) in the penultimate exon of ATP2B2. In more detail, the E1010K and F878S mutations were predicted to affect the Ca^2+^-binding region by changing local interactions with neighboring amino acids. Indeed, their proximity to the Ca^2+^-binding triad (E457, D914, and D918) suggests that both the charge substitution (E1010K) and the loss of the aromatic side chain (F878S) could impact the pump’s capacity to bind Ca^2+^. Additionally, the other substitutions were in structurally significant motifs: the M837I variant seems to potentially influence the phosphorylation of the catalytic aspartate, whereas the G120R substitution may impact the autoinhibitory interaction with the C-terminal tail and/or the accessibility of the Ca^2+^ transport site by hindering the displacement of the calmodulin-binding autoinhibitory domain from the catalytic core. Instead, the two frameshift variants were predicted to deleteriously affect functional calmodulin-binding domain. In cell-based studies, all these variants caused changes in cytosolic Ca²⁺ handling with both loss and gain effects. The strongest effects of pump activity reduction were observed with the expression of the mutants T1086fs and F878S. Instead, the M837I and E1010K mutations slightly decreased pumping activity, while G120R and V1113fs increased it [137] (Fig. 4).

The first link between PMCA3 defects and cerebella ataxia was established by an X-exome sequencing approach that enabled the discovery of the disease-causing G1107D mutation in the *ATP2B3* gene in a family with X-linked congenital cerebella ataxia [138, 139] and later in another family in which the maternal grandfather and his two grandsons exhibited ataxia and dysarthria [140]. Such residue is located into the CaM-BD located into the C-terminal tail of the pump that is crucial for both the maintenance of the pump in an auto-inhibited state in resting condition and to allow the binding of CaM, thus restoring the pump activity. Interestingly, the expression of the mutated G1107D PMCA3 pump in model cells was shown to be responsible of both a decrease Ca^2+^ extrusion capability of PMCA3 under conditions of maximal stimulation, an impaired capability of the pump to counteract the Ca^2+^ influx from the capacitative Ca^2+^ channels, and an impaired autoinhibition mechanism in its resting state. All these data suggested that the prolonged retention of Ca^2+^ within the cytosol could be the causative factor of the disease [138, 139]. Such effects were shown to be caused by the fact that in resting condition the G1107D replacement strongly reduced the interaction between the CaM-BD with both CaM and the pump core [141]. After the abovementioned report establishing a direct link between PMCA3 mutation and cerebellar ataxia, several polymorphisms emerged in patients with the same disease accompanied by a broad spectrum of additional neurological disorders ranging from neurodevelopmental disorders, dystonia, mental retardation, behavioral symptoms or seizures. Among them, into the C-terminal tail other two substitutions were discovered: the E1081Q and the R1133Q missense mutations located immediately upstream and after the CaM-BD of the pump, respectively. Interestingly, the E1081Q substitution was found in two unrelated patients suffering from neurodevelopmental (SCN1A) or neurodegenerative disorders (IBA57) and cerebellar dysfunction, while the R1133Q PMCA3 variant was discovered in a patient with a *SLC2A1* gene polymorphism and affected by neurodevelopmental delay and moderate intellectual disability [142]. However, of the two mutations reported above, only the E1081Q was shown to be responsible for profound alterations in the Ca^2+^ extrusion activity of the pump, although such effect was shown to occur in an isoform-dependent manner. Indeed, while in the full-length *b* variant such mutation is responsible for decreasing the ability of the pump to reduce [Ca^2+^] in the sub-PM (as the G1170 mutation), the same mutation inserted into the truncated *a* variant was shown to increase the Ca²⁺ extrusion activity of the pump in the sub-PM Ca^2+^ microdomains, thus highlighting the different role of PMCA splicing variants in the regulation sub-PM microdomains of [Ca^2+^]. Such differences might be brought about variations in their Ca^2+^ binding capacity, which would hamper allosteric regulation, or by different partners of the two variants that may regulate differently their activity. However, the above-cited mutations located in the C-terminal tail of PMCA3 were not the unique ones found to be related to ataxia. In fact, in the second cytosolic loop between the fourth and fifth transmembrane domains, several function-affected mutations were found to be associated with the pathology. In particular, the R482H replacement found in an ataxic patient with developmental delay and hypotonia was reported in model cells to impair the Ca^2+^ extrusion function of the pump both in stimulated cells or in resting condition [143]. Probably, such effect is due to a structural change that destabilize the portion of the pump surrounding the mutated residue, i.e., by increasing the distance with the catalytic D530 amino acid, in the Ca^2+^-bound state. Taking into consideration that the PMCA3 R482H mutation co-occurs with a compound heterozygous mutation in the *LAMA1* gene coding for the laminin subunit 1α (already demonstrated to be linked to cerebellar dysplasia [144]), it is reasonable to suggest that PMCA3 dysfunction may act as a digenic modulator in Ca^2+^-linked pathologies, thus contributing to, or exacerbating, ataxic symptoms related to *LAMA1* mutations [144]. Also, the G733R mutation in the catalytic P-domain of the PMCA 3 pump was reported in a patient affected by non-progressive ataxia, muscular hypotonia, dysmetria and nystagmus [145]. Its expression in model cells demonstrated that such mutation was responsible for partially compromised activity of the pump into the handling of Ca^2+^ rises upon Ca^2+^ release from the ER or influx through the PM, although the expression level or subcellular localization and without affecting pump expression or subcellular targeting thus causing a sustained cytosolic accumulation of Ca^2+^ [145]. By in silico modelling, it was possible to suggest that the G733R substitution could be responsible for the perturbation of local structure, and the displacement of the loop that begins with K764. Also in this case, the G733R mutation coexisted with the two R123Q and G214S heterozygous mutations in the gene encoding the enzyme phosphomannomutase 2 (PMM2), responsible of the catalyzation of mannose-6-phosphate isomerization to mannose-1-phosphate, a precursor of GDP-mannose. PMM2 mutations have already been associated with congenital multiorgan disorders related to glycosylation [146]. The coexistence of these mutations in PMCA3 and PMM2 is of particular importance as PMM2 is a Ca^2+−^regulated enzyme [147], suggesting that mutant PMCA3 may increase Ca²⁺ concentration in the microdomains where PMM2 is located, thereby inhibiting it. This, together with PMM2 mutations, would enhance the decline of PMM2 enzyme activity, contributing to the development of the ataxia phenotype. In the same loop, more recently, another mutation was discovered. In the same loop, in the stalk region of the pump upstream the P-domain, the R696H mutation was found in a patient whose clinal phenotype was related to cerebellar ataxia, although such replacement failed to cause global changes in the PMCA3 Ca^2+^ extrusion activity. However, in this case, no clinical data were available and further analysis revealed that the variant represents a rare polymorphism in line with the absence of effect on Ca²⁺ pumping defects [142]. The role of PMCA3 mutations in ataxia-related pathologies was further supported by the discovery of the spontaneous R35C PMCA3 mutation in a X-linked recessive rat model of progressive PC degeneration exhibiting a shaking ataxia and wide stance. However, no difference in the distribution or abundance of the pump was detected in PCs and biochemical studies in cell models in which the mutant pump was overexpressed showed no significant difference in its activity compared to the WT counterpart thus suggesting that such mutation is not causative, but very closely linked to the causative still not unveil *shaker* mutation [148] (Fig. 4). The aforementioned results highlight the concept that PMCA3 pump, also in specialized cells like neurons, does not have a quantitatively significant role in the global control of cellular Ca^2+^ homeostasis. However, increase or decrease in the temporal cytosolic calcium oscillations is of paramount importance for the regulation of Ca^2+^ signaling in selective sub-plasma membrane microdomains, which may be significantly impacted by the mutations even if the measurement of global Ca^2+^ changes in the cell only shows minor effects. Such microdomains host a number of proteins/enzymes regulated by Ca^2+^ and PMCA3 could regulate their activity in a way that, also beyond the detection levels of the experiment performed in cell models, would be sufficient to generated neuronal dysfunction. Evidently, the tight control of Ca^2+^ signaling by the PMCA2 and PMCA3 pumps is essential to sustain the correct functioning of the cerebellar micro and long range-circuitry.

### So, why PMCA? And why cerebellar ataxia? Future perspectives

Previous studies conducted in PMCA2 knockout PMCA2(-/-) mice, or by pharmacologically inhibiting PMCA activity, demonstrated an enhanced synaptic GABAergic inhibition within the molecular layer (ML) of cerebellar cortex. This was associated with an increased frequency and amplitude of spontaneous inhibitory post-synaptic currents and an elevated spontaneous firing rate of ML interneurons, leading to slower PN firing in PMCA2(-/-) mice and likely contributing to their ataxic phenotype [149, 150]. A striking feature of PMCA biology in the cerebellar cortex is that its expression is not uniform across synaptic inputs (Fig. 2). At PCs, the two major excitatory pathways PFs and CFs differ not only in their presynaptic release properties but also in their postsynaptic calcium-handling machinery. PF–PC synapses are characterized by low probability of release and strong short-term facilitation [151], whereas CF inputs exhibit high release probability, paired-pulse depression, and powerful, all-or-none postsynaptic responses [152]. These differences place distinct demands on the temporal profile of postsynaptic calcium transients. In PF synapses, where many weak inputs must be integrated to influence PC output, calcium signals must be carefully tuned: brief enough to avoid saturation or excitotoxicity, but sufficiently prolonged to enable summation and trigger plasticity-related signaling. By contrast, CF inputs deliver large calcium influxes in a single event, such that even rapid clearance does not prevent the robust activation of signaling cascades. Although most studies have examined PF synapses, the precise pre- and postsynaptic subtypes of PMCAs at CF synapses have yet to be determined (Fig. 2). What is the physiological role of the PMCAs at the CFs? Could mutations alter these synaptic functions at the network level? In this context, the relatively low expression of PMCAs at PF synapses likely reflects an adaptive specialization. Reduced calcium clearance by PMCA allows PF-driven Ca²⁺ transients to persist long enough to engage molecular pathways required for long-term potentiation (LTP) and long-term depression (LTD). This slower decay may also facilitate the temporal integration of repeated PF inputs, allowing PCs to decode the frequency and timing of PF activity more effectively. In contrast, CF synapses, with their massive glutamatergic input and strong postsynaptic depolarization, can tolerate higher PMCA activity without compromising plasticity, because the initial calcium influx is already large and prolonged. Thus, differential PMCA expression shapes the spatial and temporal dynamics of Ca²⁺ signaling across input types, enabling PF and CF synapses to support distinct forms of plasticity. Crucially, this specialization creates a vulnerability: if PMCA activity at PF synapses is either too low or too high, the system may fail. Insufficient clearance could cause excessive calcium accumulation, dysregulating LTD/LTP balance and risking excitotoxic stress. Conversely, hyperactive PMCA could truncate PF-driven Ca²⁺ signals before they effectively recruit second messenger pathways, impairing synaptic integration and plasticity. Both scenarios would disrupt the fine-tuned coordination between PF and CF inputs that is essential for cerebellar learning and motor control. A similar scenario can be envisioned for synaptic short- and long-term plasticity which are also highly dependent on the timely regulation of Ca^2+^ homeostasis at pre- and postsynaptic level: sustained elevations in presynaptic calcium levels may potentiate neurotransmitter release, thereby altering the mechanisms underlying synaptic plasticity and promoting excessive summation of synaptic responses. Conversely, shorter calcium transients or insufficient calcium elevations may compromise information processing by weakening synaptic efficacy. Genetic studies in both patients and experimental models have shown that PMCA mutations, whether leading to loss or gain of function, can produce ataxia, underscoring the physiological importance of maintaining PMCA activity within a narrow optimal range. Further supporting the critical role of PMCA in synaptic function, recent findings have identified its presence within a presynaptic triad composed of Cav2/Cav1/PMCA, where PMCA enables the independent control of synaptic vesicle (SV) release and recycling rates [29]. We propose that dysregulation of PMCA activity represents a convergent mechanism that can underlie cerebellar ataxia, whether through loss or gain of function. PMCA actively extrudes Ca²⁺ from the cytosol to the extracellular space. Both temporal insufficient and excessive PMCA activity can be deleterious for neuronal signaling. On the one hand, reduced PMCA function may allow cytosolic Ca²⁺ to accumulate, prolonging calcium transients to unwanted levels and perturbing activity-dependent signaling cascades. On the other hand, excessive PMCA activity may clear calcium levels too rapidly, truncating signals required for the activation of downstream messengers, while also potentially affecting neuronal membrane polarization due to altered ionic gradients. Thus, both extremes of PMCA regulation could disrupt the precise spatiotemporal patterns of calcium signaling (Fig. 3) that are essential for synaptic plasticity, ultimately impairing cerebellar information processing and motor coordination.

Taken together, these considerations suggest that PMCA is not merely a housekeeping pump but a circuit-specific regulator of calcium signaling, whose expression patterns and activity levels are critical for shaping the rules of synaptic plasticity. At PF synapses, lower PMCA levels extend calcium signals in a way that supports the integration of weak, facilitating inputs, whereas at CF synapses, stronger inputs ensure robust plasticity despite higher clearance capacity. Further experiments and evidence may help elucidate the specific roles and localization of PMCA subtypes at PF and CF synapses under physiological and pathological conditions. We hypothesize that disruption of this balance (whether through genetic mutation, altered expression, or maladaptive regulation) impairs synaptic plasticity and PC output, thereby contributing to the pathophysiology of cerebellar ataxia.

## Conclusions

Plasma Membrane Ca²⁺ ATPases, particularly the neuronal isoforms PMCA2 and PMCA3, emerge as key regulators of calcium homeostasis in PCs and, more broadly, in the cerebellar cortex. Their high-affinity function enables the fine control of Ca²⁺ microdomains, which is essential for balancing synaptic plasticity with protection against excitotoxicity. Clinical and experimental evidence demonstrates that mutations in these transporters are directly linked to forms of cerebellar ataxia, underscoring their role not merely as “housekeeping pumps” but as true circuit-specific modulators.

Looking ahead, it will be crucial to understand how the different isoforms and splice variants cooperate within distinct synaptic subdomains, and how their dynamic regulation influences long-term plasticity and connectivity. Integrative approaches combining genetics, functional imaging, and animal models may help determine whether pharmacological modulation of PMCAs can be translated into novel therapeutic strategies for ataxias and other neurodegenerative disorders.

## Data Availability

No datasets were generated or analysed during the current study.
